# Neonatal Lupus Erythematosus

**DOI:** 10.1155/2012/301274

**Published:** 2012-09-02

**Authors:** Kam Lun Hon, Alexander K. C. Leung

**Affiliations:** ^1^Department of Pediatrics, The Chinese University of Hong Kong, Prince of Wales Hospital, 6/F, Clinical Sciences Building, Shatin, Hong Kong; ^2^Department of Pediatrics, Alberta Children's Hospital, The University of Calgary, Calgary, AB, Canada T2N 1N4

## Abstract

Neonatal lupus erythematosus (NLE) refers to a clinical spectrum of cutaneous, cardiac, and systemic abnormalities observed in newborn infants whose mothers have autoantibodies against Ro/SSA and La/SSB. The condition is rare and usually benign and self-limited but sometimes may be associated with serious sequelae. We review the pathophysiology, clinical features, and management of infants with this condition. Neonates with NLE should be managed at a tertiary care center. Multidisciplinary team involvement may also be indicated. In mothers with anti-Ro/SSA and/or anti-La/SSB antibodies and infants with congenital heart block, the risk of recurrence in subsequent offspring is 17–25%. Therefore, careful monitoring of subsequent pregnancies with serial ultrasonography and echocardiography is essential.

## 1. Introduction

Neonatal lupus erythematosus (NLE) refers to a clinical spectrum of cutaneous, cardiac, and systemic abnormalities observed in newborn infants whose mothers have autoantibodies against Ro/SSA, La/SSB, and, less commonly, U1-ribonucleoprotein (U1-RNP) [1–3]. The condition was first described in 1954 by McCuistion and Schoch who reported a case of transient lupus skin lesion in an infant with an ANA-positive mother [4]. The most common presentation is a nonscarring, nonatrophic skin lesion which resemble subacute cutaneous lupus erythematosus. The infants may have no skin lesions at birth but develop them during the first weeks of life. Cardiac, hematological, hepatobiliary, central nervous, and pulmonary systems may also be involved. NLE is associated with transplacental passage of autoantibodies such as anti-RoSSA and anti-La/SSB [5, 6]. The condition is usually benign and self-limited but sometimes may be associated with serious sequelae. 

## 2. Pathophysiology

A number of studies have suggested that NLE is caused by the transplacental passage of maternal autoantibodies [5, 7]. These autoantibodies may cause damage to the developing tissue and increase the risk of bearing infants with NLE. Approximately 98% of affected infants have maternal transfer of autoantibodies against Ro/SSA, La/SSB, and, less commonly, U1-RNP. However, only 1-2% of mothers with these autoantibodies have neonates with NLE, regardless of whether the mothers are symptomatic or not [8].

The 52-kD Ro/SSA (Ro52) ribonucleoprotein is an antigenic target strongly linked with the autoimmune response in mothers whose children have NLE, congenital heart block, and other conduction abnormalities [9]. Anti-Ro52/SSA autoantibodies antagonize the serotonin-induced L-type calcium channel activation on human fetal atrial cells and trigger an inflammatory response, leading ultimately to fibrosis and scarring of the atrioventricular node, sinus node, and His bundle [9, 10]. This may explain the electrophysiological abnormalities in NLE and the pathogenesis of the cardiac rhythm disturbances, which may lead to diminished cardiac output and the subsequent development of congestive heart failure [9]. In a rat model, Boutjdir et al. [11] demonstrated that IgG containing anti-Ro/SSA and -La/SSB antibodies induces complete AV block in beating hearts and in multicellular preparations, thus implicating a preferential interaction of these autoantibodies with calcium channels and/or associated regulatory proteins. This is consistent with the observed inhibition of calcium channels that may be a critical factor contributing to the pathogenesis of complete heart block. These conduction defects are caused by anti-Ro/SSA and anti-La/SSB antibodies as well as other autoantibodies against cardiac adrenoceptors and muscarinic acetylcholine receptors [12].

The antibodies associated with heart block and with cutaneous disease are believed to be different; antibodies against the 52/60-kD Ro/SSA and 48-kD La/SSB ribonucleoproteins are associated with heart block, whereas antibodies against the 50-kD La/SSB ribonucleoprotein are associated with cutaneous disease [12, 13].

On the other hand, anti-U1RNP autoantibodies are usually associated with atypical cutaneous lesions without cardiac or systemic abnormalities in a small number of NLE cases and may play a role in the pathogenesis of thrombocytopenia [10]. It has been demonstrated that the anti-U1RNP antibody from patients with connective tissue disease can directly recognize a variety of antigens on the endothelial surface of the pulmonary artery, including the components of U1RNP or other unknown polypeptides. These results suggest that binding to HPAECs of this autoantibody may be one of the triggers of endothelial cell inflammation in various connective tissue diseases [14]. The spectrum of cutaneous disease in U1RNP antibody-positive infants is similar to Ro/SSA antibody-positive infants with NLE. Complete heart block was not a feature of U1RNP antibody-positive NLE. HLA typing studies show a more diverse immunogenetic pattern in U1RNP antibody-positive mothers of infants with NLE compared with Ro/SSA antibody-positive mothers.

It has been shown that the amount of maternal antibodies, rather than their presence, is associated with fetal tissue injury [13]. However, only some neonates exposed to these antibodies develop complications. Therefore, other factors such as titers of maternal antibodies, genetic predisposition, and environmental factors such as viral infection may be involved. Additionally, induction of apoptosis in cultured cardiomyocytes has been demonstrated to result in the expression of Ro/La antigens on the cell surface for recognition by circulating maternal antibodies [15]. It is speculated that *in vivo* such opsonized apoptotic cardiocytes promote an inflammatory response by resident macrophages with damage to surrounding conducting tissue.

In addition to its presence in the skin and heart, the Ro antigen is also found in the liver, bowel, lungs, brain, and blood cells—the tissues that are most often affected by NLE [3]. Ultraviolet radiation and estrogens increase Ro antigen expression on the surface of the keratinocyte [3]. Although ultraviolet radiation can induce or exacerbate the skin lesions, it is not required for their development [10]. Because of limited opportunity for solar exposure in neonates and young infants, photosensitivity is more commonly seen after phototherapy for neonatal hyperbilirubinemia [10].

## 3. Epidemiology

NLE is a rare acquired autoimmune disease that occurs in 1 of every 20,000 live births in the USA [5]. Elsewhere, epidemiology is usually described in small case series [7, 16]. The presence of certain major histocompatibility complexes such as human leukocyte antigen B8 and human leukocyte antigen DR3 in the mother predisposes the infant to NLE and congenital heart block [17, 18]. Although there is no apparent racial predilection, disparity in outcomes between minorities and whites has been observed [5, 10, 16, 18–20]. Like many autoimmune diseases, reports from the Research Registry for Neonatal Lupus/US indicate that the female-to-male ratio is approximately 2 : 1 with cutaneous NLE, but the gender distribution for cardiac disease is approximately equal [21, 22]. The risk of NLE or congenital heart block developing in a woman who tests positive for Ro/SSA who has never had a child with NLE or congenital heart block is less than 1%. Many seropositive mothers with anti-Ro/SSA and anti-La/SSB antibodies give birth to infants who do not show signs and symptoms of NLE. However, in those who have had an infant with NLE, the risk of cardiac and/or skin disease for a future pregnancy is high. The incidence of congenital heart block is 15–30% in infants with NLE [19]. Heart block usually develops *in utero* between the 18th and 24th weeks of pregnancy. Infants born to mothers with hypothyroidism due to thyroid autoantibodies and anti-Ro/SSA positivity are at nine times higher risk of developing congenital complete heart block than infants born to mothers with only anti-Ro/SSA positivity [23].

Approximately 40–60% of mothers are asymptomatic when the infants are diagnosed to have NLE [8]. The remaining mothers may have SLE, Sjögren syndrome, rheumatoid arthritis, or undifferentiated autoimmune disorder. Mothers with primary Sjögren syndrome or undifferentiated autoimmune syndrome have a greater risk of delivering an infant with congenital complete heart block than those with SLE [12, 24]. There is no association with paternal autoimmune diseases [12].

## 4. Clinical Manifestations

The most common clinical manifestations of NLE are, in decreasing order of frequency, dermatologic, cardiac, and hepatic abnormalities [1, 5, 10, 16, 25]. Some infants may also have hematologic, neurologic, or splenic abnormalities [5, 7, 10, 16]. One or more systems may be involved. Wisuthsarewong et al. performed a retrospective study to review clinical manifestations on 17 patients (10 girls and 7 boys) with NLE seen at the Department of Pediatrics, Siriraj Hospital from 1993 to 2008 [10]. Cutaneous, cardiac, hepatobiliary, and hematological involvement was found in 70.6%, 64.7%, 52.9%, and 35.3% of infants, respectively.

Cutaneous lesions may be present at birth but often appear within the first few weeks of life [26, 27]. Annular erythematous or polycystic plaques with or without fine scales characterize NLE and appear predominately on the scalp, neck, or face (typically periorbital in distribution), but similar plaques may appear on the trunk or extremities [10, 26]. The dermatitis resembles the rash of subacute cutaneous lupus erythematosus rather than the malar rash of SLE [25]. Periorbital erythema, referred to as “raccoon eye” or “owl eye,” is a very common characteristic [3, 10]. At times, the lesions may be urticarial, desquamative, ulcerative, or crusted [28, 29]. Bullous lesions may be seen with a particular predilection for the soles of the feet [25].

In one study, cutaneous involvement was characterized as erythematous patches (91.7%), subacute cutaneous lupus erythematosus lesions (50%), petechiae (41.7%), persistent cutis marmorata (16.7%), and discoidal lesions (8.3%) [10]. In some infants, solar exposure seems to precipitate the eruption [30]. These lesions typically last for weeks or months and then resolve spontaneously consequent to the disappearance of maternal antibodies in the neonatal circulation [26]. Active erythematous lesions after the first year of life should be suspect. Dyspigmentation is frequent but usually resolves spontaneously. Atrophic lesions and, rarely, atrophic scars may develop [10, 27]. Telangiectasia is often prominent and is the sole cutaneous manifestation reported in some patients. The atrophic telangiectatic changes are most evident near the temples and scalp and do not necessarily occur in the same sites as the erythematous lesions [26]. The latter site may occasionally be associated with a permanent alopecia. Telangiectasia, scarring, and atrophic changes are expected to persist.

The cardiac manifestations include conduction abnormalities (first-, second-, and third-degree heart block) and cardiomyopathy [1, 2, 5, 24, 31]. Third-degree heart block, once established, is usually irreversible [26]. Congenital heart block may present as bradycardia noted *in utero* or during physical examination at birth [24]. Conduction disturbances may also present as irregular heartbeat and prolongation of the QT interval [24]. Congenital heart block may be associated with endocardial fibroelastosis and cardiomyopathy [32]. In some cases, myocarditis and pericarditis can develop which may lead to bradycardia. Heart failure is a well-recognized complication during the neonatal period.

The clinical pictures of hepatobiliary involvement may take the forms of elevation of liver enzymes (such as aspartate aminotransferase and alanine aminotransferase) and/or conjugated hyperbilirubinemia occurring a few weeks or months after birth and resolving thereafter. Some infants may have mild hepatomegaly and, less commonly, splenomegaly [25]. The hepatomegaly and splenomegaly are usually transient. Cholestatic hepatitis and hepatic failure may also occur.

Hematologic disturbances (e.g., hemolytic anemia, thrombocytopenia, and neutropenia) may occur in the first 2 weeks of life. Infants with hematological involvement are usually asymptomatic [25]. Autoantibodies, mainly anti-Ro, bind directly to the neutrophil and cause neutropenia. Thrombocytopenia may manifest as petechiae. Hematologic symptoms usually appear at around the second week of life and disappear by the end of the second month. Lymphopenia is a relatively common finding in adults with SLE but is not a characteristic hematologic abnormality of NLE [26].

Other abnormalities such as hydrocephalus and macrocephaly may occur [33]. Aseptic meningitis and myelopathy have rarely been reported [10]. Pneumonitis may manifest as tachypnea and/or tachycardia.

## 5. Diagnosis and Differential Diagnosis

The diagnosis is usually established based on the clinical features and the demonstration of NLE-associated antibodies in the serum of the mother or the affected infant [5, 10, 16]. NLE can mimic many conditions [5, 10, 16]. Differential diagnosis of NLE includes seborrheic dermatitis, atopic dermatitis, neonatal acne, tinea corporis, psoriasis, granuloma annulare, erythema multiforme, Langerhans cell histiocytosis, congenital rubella, congenital syphilis, Bloom syndrome, and Rothmund-Thomson syndrome [3].

## 6. Laboratory Investigations

NLE is associated with the anti-Ro/SSA antibody in more than 90% of patients [9]. Occasionally, patients only have anti-La/SSB or anti-U1RNP antibodies. Screening of infants with NLE for the presence of these antibodies is strongly recommended [2]. Many asymptomatic mothers have positive putative antibodies during pregnancy [10]. As such, these mothers of patients suspected of having neonatal lupus erythematosus should be screened for antinuclear, anti-double-stranded DNA, anti-Ro/SSA, anti-La/SSB, and anti-U1-RNP antibodies, irrespective of their symptoms or clinical status [9]. As anti-Ro/SSA antibodies can be detected in one in 200 pregnant women, the risk for an anti-Ro/SSA-positive woman to have an infant with NLE is relatively low [26]. On the other hand, high anti-Ro/SSA levels correlate with the risk of cardiac complications. Serial prenatal ultrasonography/electrocardiography should therefore be performed on pregnant women with high anti-Ro titers (≥50 U/mL) [13]. Prenatal ultrasonography may help identify NLE that affects the heart. Echocardiography may reveal various types of structural deformities in the heart; combined electrocardiography and 24-hour Holter monitoring may reveal various cardiac conduction disorders or different types of heart blocks.

Laboratory investigations may reveal pancytopenia, thrombocytopenia, leukopenia, or elevated transaminase level [34].

Skin biopsy is useful in patients with NLE when the diagnosis is in doubt. Histologic examination shows interface dermatitis, keratinocyte damage, moderate hyperkeratosis, follicular plugging, and vacuolar degeneration in the basal cell layer. Epidermal atrophy may be found [26]. Inflammatory infiltrate may be intense with bulla formation histologically. An immunofluorescent examination reveals a granular deposition of immunoglobulin G (IgG) at the dermoepidermal junction; IgM and C3 deposition may also be evident.

Skin biopsy is not pathognomonic. Various inflammatory and infectious conditions may show similar histological features. In typical cases of NLE and positive autoantibodies, skin biopsy is not mandatory to confirm the diagnosis.

## 7. Treatment and Followup

Neonates with NLE should be managed at a tertiary care center. Multidisciplinary team involvement may also be indicated. Patients with NLE with cardiac involvement require regular monitoring to assess cardiac function and the need for a pacemaker. A pacemaker is often necessary for those who are unable to compensate for a slow heart rate. Serial echocardiography to monitor for a prolonged PR interval should also be arranged. If the cardiac involvement is severe, activity may have to be restricted in the young child.

Sunscreens may be useful in the treatment of cutaneous lupus erythematosus, but a neonate is less likely to be exposed to sunlight excessively. Nevertheless, solar exposure should be avoided if possible. Parents should be advised to apply sunscreen well before solar exposure and to use a sunscreen with a high SPF that provides a broad-spectrum (UV-A) coverage which is water resistant. Behavior modification to include solar avoidance should be encouraged. Protective clothing is highly desirable. Strategies aimed at preventing disease before irrevocable scarring ensues are a high priority. Skin lesions of NLE can be treated with mild topical corticosteroids. Antimalarial agents have potential toxicity and a slow onset of action that their use in the treatment of this transient condition is probably not indicated [26]. Laser therapy may be considered for residual telangiectasia. Systemic corticosteroids and immunosuppressive agents are generally not indicated in the treatment of NLE [26]. Children with NLE need continued followup, especially before adolescence and if the mother herself has an autoimmune disease [35]. Although the child may not be at increased risk of developing SLE, the development of some form of autoimmune disease in early childhood may be of concern.

Infants with severe hepatic and hematological involvement may require treatment with systemic corticosteroids, intravenous immunoglobulin, and/or immunosuppressive agents [10].

## 8. Prognosis

The morbidity and mortality of SLE of childhood depend on the organ systems affected [5, 7]. Children with NLE have an excellent long-term outcome when only skin lesions are present [36]. The cutaneous lesions usually disappear by 6 months of age coincident with the clearance of maternal antibodies from the child's circulation [5, 24, 31]. Involvement of the skin may, rarely, lead to scar formation. Although children with cutaneous disease may be more prone to develop SLE or autoimmunity later in life, this is mainly due to their genetic predisposition, not that they had NLE. Their nonaffected siblings are also at risk for development of SLE or autoimmunity. While the cutaneous lesions of NLE are themselves benign, cutaneous NLE is associated with a 6–10-fold risk for a subsequent child with cardiac NLE [5, 24, 31].

NLE with cardiac involvement is associated with a 20–30% mortality rate in the neonatal period [5, 24, 31]. Mortality is particularly high in cases of congenital heart block with concurrent cardiomyopathy [19, 26]. Death most often results from congestive heart failure caused by congenital heart block. Approximately 57 to 66% of patients with congenital heart block eventually require a pacemaker [5, 24, 31, 36]. Those with pacemakers are at risk of developing dilated cardiomyopathy in their lives [37]. Deaths may also occur later in life as a result of the failure of the pacemaker. However, many children with congenital heart block may be relatively asymptomatic until adolescence, when they begin to exercise. At that time, they may develop syncope and require a pacemaker implantation.

The recurrence rate of congenital heart block is low, about 15%, but this is nearly three times higher than the risk for congenital heart block in a primigravida with the putative antibodies [12]. Prospective clinical trials on use of antenatal fluorinated steroids in women with anti-SSA/Ro and/or anti-SSB/La antibodies and fetuses with heart block identified *in utero* are required before definitive recommendations can be made. A number of anecdotal cases support the use of dexamethasone for treatment of hydrops and possibly incomplete block [12]. 

Most patients with NLE affecting liver or blood have transient disease that spontaneously resolves within 4–6 months. In some cases, cholestatic hepatitis and liver failure may occur which is associated with a poor prognosis. Anemia, thrombocytopenia, and neutropenia are self-limited. However, if severe thrombocytopenia is present, internal bleeding can lead to a poor prognosis.

## 9. Future Pregnancies

Although the fetal disease is called neonatal lupus erythematosus, this is considered a misnomer since only about 25% of mothers actually fulfill criteria for the diagnosis of SLE [12]. Furthermore, asymptomatic mothers do not invariably become ill [12]. Mothers of infants with NLE, particularly infants with congenital heart block, have a 2-fold to 3-fold increased risk of having an affected infant in a subsequent pregnancy. On the other hand, the risk for an unselected anti-Ro/SSA-positive woman has been estimated at 1-2% [26]. A prospective controlled study of pregnancy outcome in 100 women with autoimmune diseases and anti-Ro/SSA antibodies showed that the prevalence of congenital heart block in newborns of prospectively followed up women already known to be anti-Ro/SSA positive and with known connective tissue disorders was 2% [24, 38].

In mothers with anti-Ro/SSA and/or anti-La/SSB antibodies and infants with congenital heart block, the risk of recurrence in subsequent offspring is 17–25% [2, 39]. Therefore, carefully monitoring of subsequent pregnancies with serial ultrasonography and echocardiography, particularly at 18–24 weeks' gestation, is essential. Intravenous immunoglobulin merits evaluation as a potential prophylactic approach in mothers who have previously had an affected child [40]. However, two studies failed to demonstrate benefit in outcome from intravenous immunoglobulin [41, 42]. On the other hand, the use of hydroxychloroquine for patients with SLE has been associated with a lower rate of NLE during pregnancy [43].

Shinohara et al. assessed the possibility of preventing cardiac or cutaneous manifestations of NLE or treating the fetus with congenital heart block by administering corticosteroid therapy to the mother [44]. Eighty seven offspring of 40 anti-Ro/SSA-positive mothers, followed up from 1979 to 1996, were evaluated. None of 26 neonates whose mothers received corticosteroid maintenance therapy initiated before 16 weeks' gestation demonstrated congenital heart block, whereas 15 of 61 neonates whose mothers received no corticosteroids during pregnancy or began receiving steroid therapy after 16 weeks' gestation had congenital heart block. Complete congenital heart block, once developed, did not respond to corticosteroid treatment *in utero*. Four infants whose mothers received corticosteroid treatment before 16 weeks' gestation had skin lesions of NLE. The authors concluded that once established, complete congenital heart block was irreversible, and maternal corticosteroid therapy did not effectively prevent cutaneous LE. However, prenatal maintenance therapy with prednisolone or betamethasone given to the mother starting early in pregnancy (before 16 weeks' gestation) might reduce the risk of developing antibody-mediated congenital heart block in the offspring [44].

Mothers with SLE should be treated with drugs that are effective and safe for the fetus [45]. Such an approach may diminish or reduce the prevalence of complete heart block associated with NLE. Tincani et al. recently reported increased occurrence of learning disabilities in children born to mothers with SLE [45]. Corticosteroids and some immunosuppressive drugs can be used in pregnancy to control maternal disease. Some data suggest that prolonged fetal exposure to dexamethasone may impair cerebral development [46]. On the other hand, Tincani et al. followed 6 children (age range, 14–65 months), born to patients treated with dexamethasone because of congenital heart block [45]. These children were found to have a normal intelligence quotient [45]. However, the authors remarked that information about long-term outcome of children exposed to immunosuppressive drugs “*in utero*” are still lacking, and more efforts are needed in this research area.

## 10. Summary

Neonatal lupus erythematosus (NLE) refers to a clinical spectrum of cutaneous, cardiac, and systemic abnormalities observed in newborn infants whose mothers have autoantibodies against Ro/SSA and La/SSB. The condition may be associated with serious sequelae. Neonates with NLE should be managed at a tertiary care center, and multidisciplinary team involvement may be indicated.
